# Xylosyltransferase-I in knee arthrofibrosis: Mechanistic insights and translational implications

**DOI:** 10.1016/j.jor.2026.02.031

**Published:** 2026-02-05

**Authors:** Yong Huang, Chao Lou, Michael Jagodzinski

**Affiliations:** a Hannover Medical School, Hannover, Germany; b Agaplesion EV. Klinikum Schaumburg, Zum Schaumburger Klinikum1, 31683, Obernkirchen, Germany

**Keywords:** Xylosyltransferase, Arthrofibrosis, Total knee arthroplasty, Wnt/ß-catenin, TGF-ß1, Biomarker

## Abstract

**Background:**

Arthrofibrosis is a frequent complication after total knee arthroplasty (TKA) and remains difficult to diagnose early due to the lack of reliable biomarkers. Excessive extracellular matrix (ECM) remodeling is driven by core signaling cascades, notably the Transforming Growth Factor-beta 1 (TGF-ß1) and Wnt/ß-catenin pathways. Xylosyltransferase-I (XT-I), a key enzyme regulating proteoglycan biosynthesis, has emerged as a critical downstream effector and potential indicator of early fibrotic activity.

**Methods:**

This narrative review summarizes clinical and experimental findings on XT-I in joint fibrosis, with emphasis on its role within the molecular network of key pro-fibrotic signaling and its diagnostic and translational potential in knee arthrofibrosis.

**Results:**

XT-I is consistently upregulated in fibrotic synovial fibroblasts and synovial fluid of arthrofibrotic knees, correlating with ECM remodeling and myofibroblast activation induced by both TGF-ß1and Wnt/ß-catenin signaling. XT-I demonstrates local rather than systemic diagnostic value and may serve as an early fibrosis indicator.

**Conclusion:**

XT-I holds promise as a synovial biomarker and potential therapeutic target in arthrofibrosis. Targeting XT-I, potentially in combination with core pathway inhibitors (e.g., Wnt/ß-catenin inhibitors), may offer a refined strategy for early diagnosis and postoperative management in TKA patients.

## 1 Introduction

**Fig. 1:**
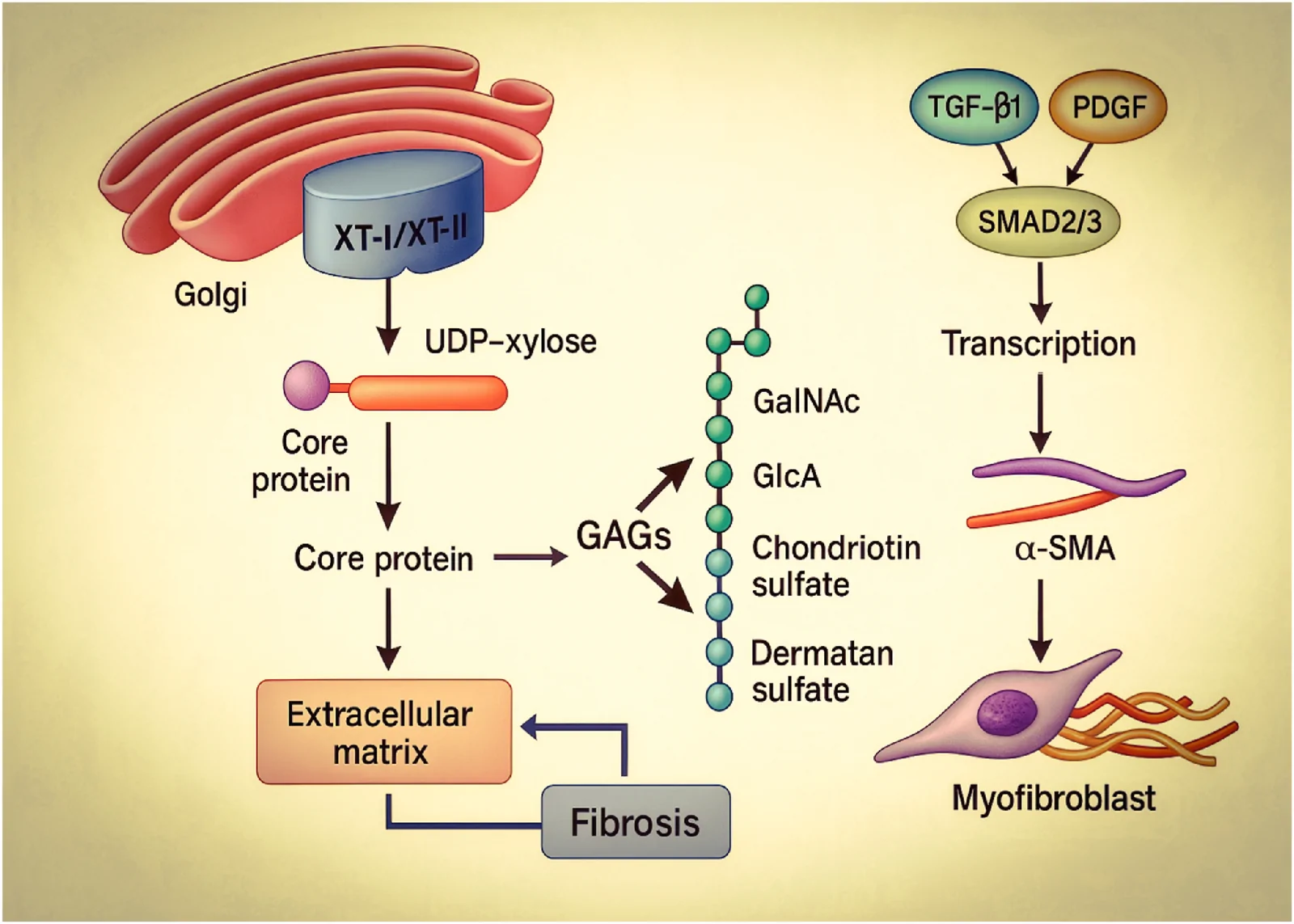
Structure and function of XT

**Fig. 2:**
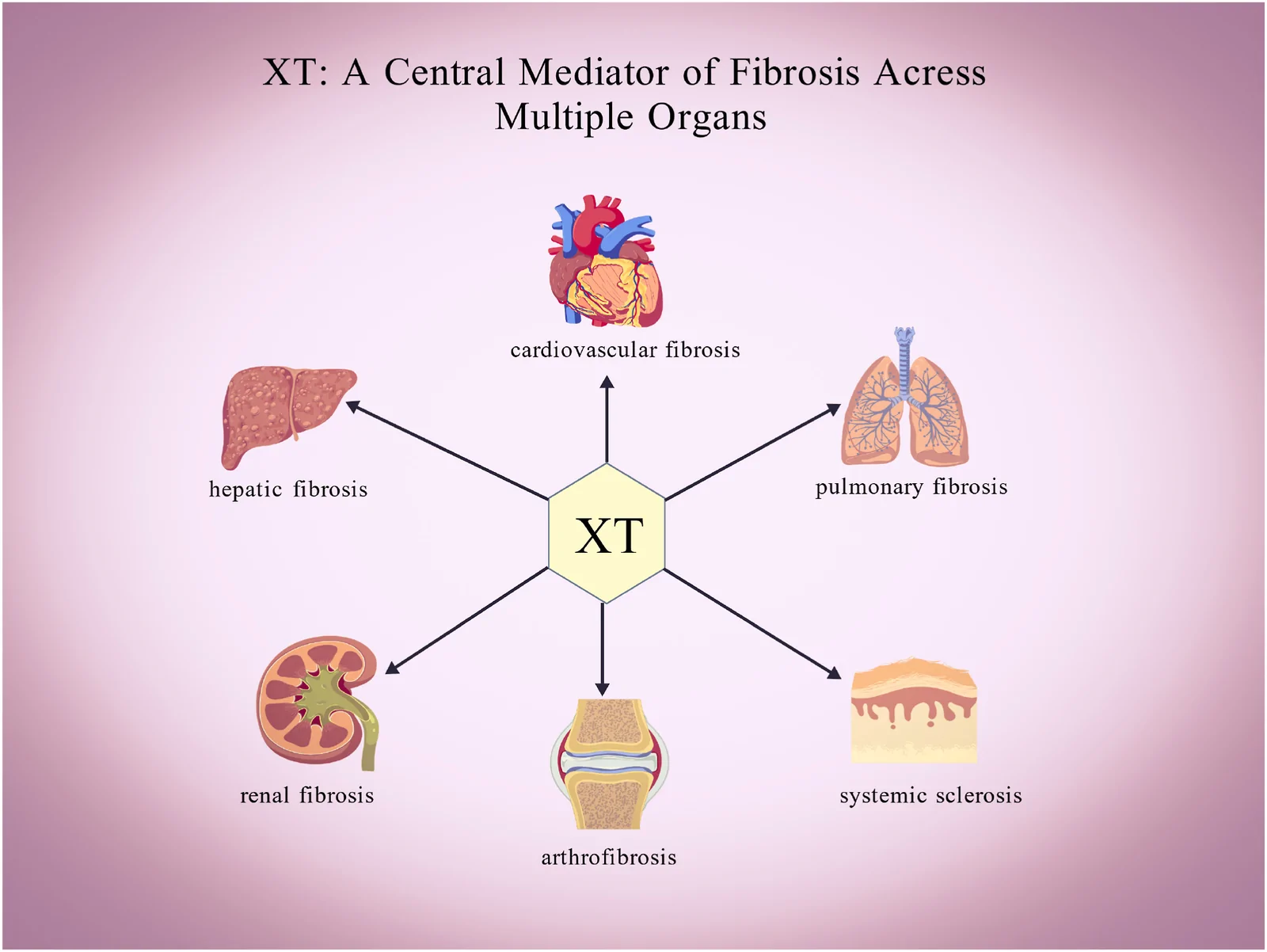
XT in multipule Fibrotic Disorders.

Total knee arthroplasty (TKA) is a widely accepted treatment for end-stage joint diseases. However, approximately 5–10% of patients develop primary arthrofibrosis postoperatively, characterized by pain, limited range of motion, and functional impairment 1, 2, 3. Despite its prevalence, the pathogenesis of arthrofibrosis remains poorly understood, with contributing factors ranging from mechanical irritation, immune dysregulation, aberrant wound healing, to genetic predisposition. Early diagnosis and timely intervention are crucial, yet currently, no sensitive or specific biomarkers are clinically validated to monitor or predict fibrotic progression.^4^^,^^5^ The lack of reliable molecular tools limits our ability to stratify patients by risk, predict therapeutic response, or evaluate treatment efficacy. Hence, there is a pressing need to identify and validate novel biomarkers that reflect fibrotic remodeling within the joint (Fig. 1, Fig. 2).

The pathological core of arthrofibrosis involves the uncontrolled remodeling of the Extracellular Matrix (ECM), primarily driven by the differentiation of Synovial Fibroblasts (SFs) into highly contractile Myofibroblasts. This pathological transition is orchestrated by a complex network of molecular signaling pathways. The Transforming Growth Factor-beta 1 (TGF-ß1) pathway is universally recognized as the central cytokine initiating the fibrotic cascade. Crucially, the canonical Wnt/ß-catenin signaling pathway has recently gained attention for its critical and synergistic role in arthrofibrosis. Studies indicate that the Wnt/ß-catenin pathway not only drives the sustained activation of myofibroblasts and ECM synthesis but also forms a positive feedback loop with the TGF-ß1pathway, collectively driving pathological scar formation. Therefore, a deeper understanding of these core signaling networks is fundamental to identifying precise therapeutic intervention points.

Xylosyltransferase-I (XT-I) is the key rate-limiting enzyme responsible for initiating the biosynthesis of Glycosaminoglycan (GAG) chains found in ECM proteoglycans. As proteoglycans are central structural components contributing to joint stiffness, XT-I activity is directly correlated with fibrotic progression. In contrast to many studies that focus on upstream signaling drivers (e.g., TGF-ß1 and Wnt/ß-catenin), XT-I represents a vital downstream enzymatic effector whose activity integrates the signals emanating from these major pro-fibrotic cascades.

This narrative review aims to provide a comprehensive summary of XT-I's role in knee arthrofibrosis. We will not only review XT-I's potential as a local diagnostic biomarker but will also strategically dissect its function as a key downstream effector within the TGF-ß1 and Wnt/ß-catenin driven fibrotic network. By bridging the knowledge gap between upstream signaling drivers and the final ECM synthesis step, we aim to offer new insights for the early diagnosis and the development of advanced, integrated therapeutic strategies for arthrofibrosis.

## 2 Structure and Function of XT

Xylosyltransferases (XT), including XT-I and XT-II, are Golgi-resident glycosyltransferases that catalyze the initial and rate-limiting step in glycosaminoglycan (GAG) biosynthesis 6, 7, 8. By transferring xylose from UDP-xylose to the serine residues of the core protein of proteoglycans, XT initiates the assembly of GAG chains, such as chondroitin sulfate and dermatan sulfate. These GAGs are essential for maintaining the viscoelastic properties of joint cartilage and modulating cellular signaling. Dysregulation of XT activity can lead to excessive accumulation of extracellular matrix (ECM), a hallmark of fibrotic disorders.

XT-I and XT-II exhibit overlapping but non-redundant roles. XT-I is primarily responsible for initiating proteoglycan biosynthesis in fibroblasts,^6^ and has been found to be significantly upregulated in fibrotic synovial tissues following joint trauma.^7^^,^^8^ In idiopathic pulmonary fibrosis, XT-I also emerged as a novel target regulating fibroblast activation,^9^ and its expression appears subject to epigenetic modulation such as promoter methylation in hypertrophic scars.^10^ Meanwhile, XT-II is believed to exert compensatory function, albeit at lower inducibility under fibrotic stimuli.

Additionally, XT enzymes modulate not only ECM deposition but also influence cellular adhesion, migration, and intercellular signaling.^8^ Recent transcriptomic studies further demonstrate that glycosaminoglycan-related enzymes—including XT-I—are overexpressed in activated synovial fibroblasts isolated from fibrotic joints, reinforcing their contribution to pathologic matrix remodeling.

## 3 Mechanistic studies of XT in arthrofibrosis and other fibrotic disorders

### 3.1 Mechanistic studies of XT in other fibrotic disorders

Beyond joint fibrosis, XT dysregulation has been documented in multiple systemic and organ-specific fibrotic conditions. In systemic sclerosis, serum XT activity is elevated and correlates with disease severity.^11^ Prospective studies indicate that patients with diffuse systemic sclerosis show up to a 2- to 3-fold increase in circulating XT activity compared with healthy controls, and XT activity tracks with modified Rodnan skin score.

Hepatic fibrosis models demonstrate that upregulated XT-I promotes glycosaminoglycan deposition in the perisinusoidal space, contributing to liver stiffness.^12^ Animal studies using carbon tetrachloride-induced liver fibrosis confirm XT-I overexpression in hepatic stellate cells and a positive correlation with Sirius Red-stained collagen area.

Similarly, pulmonary fibrosis studies reveal that TGF-β1-induced XT-I expression enhances fibroblast activation and may contribute to progressive interstitial matrix expansion.^13^^,^^14^ Bronchoalveolar lavage fluid from idiopathic pulmonary fibrosis (IPF) patients also shows elevated XT activity, which correlates with FVC decline.^15^

In renal fibrosis, glomerular and interstitial fibroblasts show increased XT-I transcription in response to chronic injury,^16^ paralleling elevated GAG synthesis and myofibroblast marker expression. In murine unilateral ureteral obstruction (UUO) models, XT inhibition reduces fibrotic scarring and collagen III deposition.

Skin fibrosis, including hypertrophic scars and keloids, also displays XT overexpression. Immunohistochemistry of keloid tissue reveals XT-I enrichment in dermal fibroblasts, and in vitro siRNA-mediated XT silencing attenuates both collagen I and decorin synthesis.^17^

Further supporting the systemic relevance of XT-I in fibrotic diseases, recent studies have confirmed its functional involvement in cardiovascular and renal fibrosis models. In the myocardium, XT-I was shown to mediate TGF-β1-induced glycosaminoglycan biosynthesis, contributing to myocardial stiffening and ventricular dysfunction in chronic heart failure models.^18^ In renal tissue, overexpression of XT-I has been associated with poor outcomes in patients with progressive glomerular sclerosis, with both diagnostic and prognostic significance.^19^ These organ-specific findings reinforce the hypothesis that XT-I acts as a shared molecular effector downstream of fibrogenic signaling cascades across diverse tissues.

In hepatic and dermal fibrosis, compounds like artesunate have been found to attenuate ECM deposition through downregulating XT-I activity via Beclin-1 mediated autophagy pathways,^20^ and XT-I itself has been subject to epigenetic control mechanisms, including DNA methylation and chromatin remodeling. These findings open new directions for therapeutic targeting and personalized intervention in tissue fibrosis.

Collectively, these data suggest that XT is a conserved mediator across fibrotic pathologies, reinforcing its candidacy as a biomarker and therapeutic target.

### 3.2 Xylosyltransferase-I (XT-I) as a TGF-ß1 target in arthrofibrosis

Transforming growth factor-beta 1 (TGF-β1) is a central cytokine in the fibrotic cascade and is known to induce myofibroblast differentiation, characterized by α-smooth muscle actin (α-SMA) expression, enhanced contractility, and increased secretion of collagen and proteoglycans.^11^^,^^21^ TGF-β1 stimulation also upregulates XT-I expression, linking this enzyme to fibrotic remodeling.

Faust et al.^1^ performed a pioneering study using primary synovial fibroblasts (SFs) from patients with arthrofibrosis, demonstrating that upon TGF-β1 exposure, XT-I mRNA expression and enzymatic activity were significantly upregulated. This correlated with elevated expression of α-SMA and COL3A1, indicating activation of the myofibroblast phenotype. Notably, XT-I activity closely mirrored the fibrotic phenotype, reinforcing its potential as a surrogate marker of disease activity. These findings were consistent across multiple donor samples.

Additionally, studies in systemic sclerosis and hepatic fibrosis have found increased XT activity correlating with fibrotic burden.^9^ In knee arthrofibrosis, where the fibrotic response is spatially localized, the tissue-specific expression pattern of XT-I renders it particularly relevant. Transcriptomic profiling of fibrotic versus non-fibrotic synovial tissues has revealed enrichment of XT-related gene networks in fibrotic samples.

Recent in vivo studies using murine models of joint fibrosis have corroborated these findings, demonstrating that knockout or pharmacologic inhibition of XT-I results in attenuated fibrotic response and improved joint mobility.

### 3.3 Wnt/ß-catenin signaling: a central driver of fibroblast activation in arthrofibrosis

The Wnt/ß-catenin canonical signaling pathway is a fundamental regulator of cellular fate and is emerging as a critical pro-fibrotic cascade in joint arthrofibrosis1. ß-catenin, the core effector of this pathway, shows sustained activation in fibrotic joint tissue, promoting myofibroblast differentiation and excessive matrix deposition.^22^ The contribution of this pathway has been documented in post-traumatic joint stiffness, scar formation after total knee arthroplasty (TKA), and the progression of inflammatory joint disease.

#### 3.3.1 The pro-fibrotic roles of ß-catenin

##### 3.3.1.1 Driving myofibroblast differentiation

Activation of ß-catenin is essential for driving synovial fibroblasts to differentiate into myofibroblasts, a hallmark cellular event of fibrosis. This is primarily characterized by the upregulated expression of -smooth muscle actin (α-SMA).^22^ Furthermore, this pathway enhances the contractility, migration, and survival of these pathological cells.

##### 3.3.1.2 Enhancing extracellular matrix (ECM) accumulation

ß-catenin activation directly upregulates the transcription of major ECM components, leading to the progressive thickening of the joint capsule and synovium. Key target genes include:

• Collagen Types: COL1A1 and COL3A1
• Adhesion/Matrix Proteins: FN1 (Fibronectin)
• Matrix Modifiers: LOX (Lysyl Oxidase), which promotes collagen cross-linking and tissue stiffness. Additionally, the pathway may contribute to ECM accumulation by inhibiting matrix-degrading enzymes such as MMP-1 and MMP-3, while potentially upregulating tissue inhibitors of metalloproteinases (TIMP family), resulting in an imbalance of matrix accumulation over degradation.^22^

#### 3.3.2 Interplay with other fibrotic pathways

The Wnt/ß-catenin pathway does not act in isolation; it forms a critical positive feedback loopwith the Transforming Growth Factor-beta 1 (TGF-ß1)/Smad signaling pathway.

• TGF-ß1 Induction: TGF-ß1, a central profibrotic cytokine known to upregulate XT-I, can also promote ß-catenin dephosphorylation and nuclear translocation
• Synergistic Enhancement: In turn, nuclear ß-catenin can enhance the transcriptional activity of Smad2/3, thereby creating a self-reinforcing fibrotic circuit.^23^ Synergistic effects have also been observed with the Hedgehog and Notch pathways in joint stiffness models, where co-activation promotes scar formation.

#### 3.3.3 Clinical and translational relevance in knee arthrofibrosis

Evidence from clinical and animal studies strongly supports ß-catenin's role:

• High Expression in Fibrotic Tissue: In murine models of knee joint fixation and capsular thickening, ß-catenin is significantly enriched in the nuclei of synovial fibroblasts and correlates with the degree of joint stiffness and collagen deposition.
• Clinical Correlation: Clinical data from patients undergoing secondary arthrolysis for stiffness have shown high expression of ß-catenin and Wnt ligands (e.g., Wnt3a) in the fibrotic synovium and capsule, correlating with the severity of limited range of motion.^24^
• Therapeutic Potential: Pharmacological inhibition of ß-catenin signaling using agents like ICG-001 (a CBP/ß-catenin inhibitor) or XAV939 (a Tankyrase inhibitor that promotes ß-catenin degradation)^25^ has been shown to significantly reduce ECM deposition, the number of -SMA positive cells, and joint capsule thickness, leading to improved joint mobility in animal models.

Integration Hypothesis: Since the Wnt/ß-catenin pathway promotes TGF-ß1 activity and both pathways synergistically drive myofibroblast activation, and XT-I is a proven TGF-ß1 target, it is strongly hypothesized that ß-catenin activation directly or indirectly upregulates XT-I expression, positioning XT-I as a crucial downstream integrator of both major fibrogenic cascades. This regulatory connection explains why targeting either upstream driver (Wnt/ß-catenin) or the downstream effector (XT-I) achieves a reduction in ECM deposition.

Collectively, the Wnt/ß-catenin pathway represents a major, druggable target whose activation profile is intricately linked with, and complements, the ECM synthesis initiated by enzymes like XT-I, offering a comprehensive strategy for anti-fibrotic intervention in arthrofibrosis.

## 4 XT as a biomarker: research progress

While XT is an intracellular enzyme, its activity can be quantified in biological fluids. Faust et al.^1^ evaluated XT-I and XT-II levels in both serum and synovial fluid of patients undergoing revision surgery for stiffness. Although serum XT levels did not significantly differ between arthrofibrosis and other causes of joint failure, XT-I concentrations in synovial fluid were markedly elevated in arthrofibrotic patients. This was accompanied by elevated levels of TGF-β1 and PDGF.

Importantly, the XT-I/XT-II ratio in synovial fluid appeared to reflect disease activity, with higher XT-I dominance correlating with increased fibrosis as per Krenn's histopathological grading.^2^ These findings suggest that local XT activity, rather than systemic, provides a more accurate reflection of fibrotic remodeling within the joint microenvironment. The feasibility of sampling synovial fluid intraoperatively or via arthrocentesis makes XT a clinically accessible candidate biomarker.

Another promising aspect is the temporal expression of XT. In vitro time-course analyses showed XT-I induction occurs early following TGF-β1 exposure, preceding peak collagen synthesis. This temporal precedence may allow XT measurement to serve as an early biomarker before irreversible fibrotic changes occur.

Furthermore, multiplex assays combining XT-I, TGF-β1, PDGF, and other matrix-associated markers could pave the way for precision diagnostics.

Beyond individual biomarker evaluation, recent studies have emphasized the value of integrating XT-I into broader multi-analyte panels. In a 2023 transcriptomic-proteomic multi-omics study of fibrotic synovial tissues, XT-I was identified as a central hub gene with high connectivity to ECM regulatory pathways, inflammatory mediators, and mechanical stress sensors.^26^ This integrative evidence highlights its potential in early fibrosis prediction, especially when analyzed alongside markers like TGF-β1, PDGF, and α-SMA.

Furthermore, machine learning approaches have started to incorporate XT-I expression patterns as part of predictive fibrosis classifiers, achieving encouraging accuracy in pilot datasets.^27^ Such advances could lead to XT-I-based composite scores for postoperative risk stratification in total knee arthroplasty patients.

## 5 Clinical applications and future perspectives

The translation of XT research into clinical practice offers multiple promising directions:

● Synovial fluid XT-I quantification could serve as a non-invasive biomarker to distinguish fibrotic stiffness from mechanical or infectious causes19.
● Combined analysis with TGF-ß1, PDGF, and Wnt pathway markers may enhance diagnostic accuracy, enabling development of a fibrosis-specific multi-analyte panel.

From a therapeutic perspective, inhibiting XT-I expression or activity, either alone or in combination with upstream pathway inhibitors, may offer novel antifibrotic strategies21. Preliminary work in fibrotic skin and liver models has demonstrated that downregulating XT reduces GAG synthesis and ECM accumulation. The development of XT-targeted therapeutics, including small molecule inhibitors, RNA-based silencing approaches, or neutralizing antibodies, represents a long-term translational goal.

Combination Therapeutic Strategies: Given that arthrofibrosis is driven by a complex network of factors including ß-catenin activation and XT-I-mediated ECM synthesis, a combination therapy approach holds significant promise. Targeting the upstream driver with Wnt/ß-catenin inhibitors (such as XAV939 or ICG-001, which have shown efficacy in reducing ECM deposition and improving joint mobility in models) while simultaneously blocking the downstream effect with an XT-I inhibitor could offer synergistic antifibrotic effects. The localized nature of knee arthrofibrosis makes the local injection of nano-carrier or hydrogel-delivered XT-I and ß-catenin inhibitors a compelling future direction for minimizing post-operative fibrosis.

XT expression may be modulated by mechanical stress and psychological stressors. Investigating such upstream regulators could uncover modifiable risk factors and pave the way for personalized rehabilitation strategies post-TKA. Integration of XT measurements into postoperative monitoring protocols could provide real-time feedback on fibrotic progression.

The development of XT-targeted therapeutics, including small molecule inhibitors, RNA-based silencing approaches, or neutralizing antibodies, represents a long-term translational goal.

In recent years, targeted inhibition of XT-I has attracted growing interest as a novel antifibrotic approach. High-throughput screening efforts have identified several small-molecule compounds capable of selectively downregulating XT-I activity in fibrotic fibroblasts without affecting basal ECM turnover.^28^ These inhibitors have shown promise in animal models of lung and skin fibrosis, reducing collagen I and GAG synthesis while preserving tissue integrity. Moreover, RNA interference and CRISPR-based gene modulation platforms are being explored to silence XT-I in joint fibroblasts, offering potential for precise local intervention.

Despite encouraging preclinical data, significant translational challenges remain. These include off-target effects, drug delivery barriers within joint environments, and inter-individual variability in XT expression. Nevertheless, early-stage pharmacologic modulation of XT-I offers a compelling future direction for minimizing arthrofibrosis following total knee replacement.

Moreover, establishing XT reference values in healthy and pathological synovial fluids, understanding inter-individual variability, and validating findings in multi-center cohorts will be essential steps toward clinical adoption.

## 6 Future research directions

Future studies should prioritize in vivo validation of XT-I as a functional driver of fibrosis and as a diagnostic marker in joint disease. Clinical trials incorporating XT-I measurement in synovial fluid during and after total knee replacement (TKR) may offer insights into its predictive value. Additionally, integrating multi-omics approaches—such as epigenetics, proteomics, and metabolomics—can help delineate the regulatory networks influencing XT-I expression and activity. Identifying upstream signals beyond TGF-β1, such as mechanical stress or inflammatory cytokines, could inform personalized treatment strategies. The development of selective XT inhibitors also warrants exploration, particularly in the context of minimizing post-operative fibrosis without impairing normal tissue repair.

Recent multi-omics studies have significantly advanced our understanding of proteoglycan structure-function relationships,^29^^,^^30^ while multicenter clinical trials are validating novel biomarker panels 31, 32, 33. These developments complement ongoing efforts to identify upstream signals beyond TGF-β1, such as mechanical stress or inflammatory cytokines, which could inform personalized treatment strategies.

In this context, multi-omics integration—including transcriptomics, epigenomics, and proteomics—offers an unprecedented opportunity to dissect the upstream and downstream regulatory networks surrounding XT-I. For instance, omics profiling of synovial tissue from arthrofibrotic patients has identified XT-I as a key regulatory node co-expressed with fibrogenic drivers such as SMAD3, LOX, and FN1.^26^ These network-level insights not only reinforce the centrality of XT-I in fibrotic remodeling but also suggest possible synergistic targets.

Furthermore, individualized XT-I expression patterns may serve as the basis for patient stratification. Integrating XT-I biomarker data into clinical algorithms and AI-based fibrosis risk models could help identify high-risk patients preoperatively or monitor early-stage fibrotic remodeling postoperatively.^27^ Such predictive capabilities could ultimately support the development of tailored therapeutic regimens and surveillance protocols.

## 7 Conclusion

Xylosyltransferase-I (XT-I) holds significant promise as a unique synovial biomarker and a potential therapeutic target in knee arthrofibrosis. XT-I is a key enzyme in the final common pathway of ECM deposition. Its expression is strongly linked to the activation of the central fibrogenic molecular network, particularly the synergistic interplay between the Transforming Growth Factor-beta 1 (TGF-ß1) and Wnt/ß-catenin signaling pathways. Integrating XT-I measurement into perioperative assessment may enhance the early diagnosis of arthrofibrosis, allowing for timely intervention. Furthermore, targeting XT-I, potentially in combination with upstream driver inhibitors such as Wnt/ß-catenin inhibitors, presents a promising strategy for developing advanced, mechanism-based treatments to effectively control post-operative joint stiffness and scar formation.

## Ethics approval and consent to participate

Not applicable. This study is a narrative review based exclusively on previously published literature and does not involve human participants, animals, or any new experimental procedures.

## Patient/guardian consent

Not applicable. This article is a narrative review based exclusively on previously published studies and does not involve any individual patient data or identifiable information.

## Credit author statement

Yong Huang: Conceptualization, Writing – Original Draft, Literature Review.

Chao Lou: Editing, Visualization.

Michael Jagodzinski: Supervision, Writing – Review & Editing, Corresponding Author.

## Funding

This work was supported by the 10.13039/501100004543China Scholarship Council (CSC).

## Declaration of competing interests

The authors declare no competing interests.

## Abbreviations

TKA: Total knee arthroplasty
ECM: Excessive extracellular matrix
TGF-ß1: Transforming Growth Factor-beta 1
XT-I: Xylosyltransferase-I
SFs: Synovial Fibroblasts
GAG: Glycosaminoglycan
IPF: Idiopathic pulmonary fibrosis
UUO: Unilateral ureteral obstruction
